# Interaction of Structured Light With Nanostructured Matter

**DOI:** 10.1002/nap2.70105

**Published:** 2026-04-30

**Authors:** Noah Apostolico, Leander Siegle, Luca Schmid, Tim‐Dominik Gómez, Julian Schwab, Mario Hentschel, Harald Giessen

**Affiliations:** ^1^ 4th Physics Institute and Research Center SCoPE University of Stuttgart Stuttgart Germany

**Keywords:** light‐matter interaction, nanophotonics, structured light

## Abstract

Although structured light has been extensively studied, its interaction with nanoscale matter has remained largely unexamined. Here, we demonstrate a symmetry‐dependent interaction between structured light and nanostructured absorbers by investigating the orbital‐angular‐momentum‐dependent extinction of gold nanodisk oligomers. Using a rigorous design framework, we fabricate diffractive optical elements via two‐photon‐polymerization grayscale lithography to generate three distinct flower‐like beam modes. Scanning these beams across oligomers of different symmetries reveals that extinction is maximized when the beam and structure share the same rotational symmetry. A semi‐analytical overlap model reproduces the observed trends with deviations consistently below 10 percentage points, confirming the robustness of the symmetry‐based interaction mechanism.

## Graphical abstract

Structured light–matter interactions at the nanoscale remain poorly understood. We reveal a symmetry‐matched mechanism governing orbital‐angular‐momentum‐dependent extinction in gold nanodisk oligomers. Tailored diffractive optical elements generate flower‐like beams whose rotational symmetry dictates absorption. Experiments and a semi‐analytical model agree, showing maximal extinction when beam and structure symmetries coincide, offering a predictive design principle for nanophotonics.

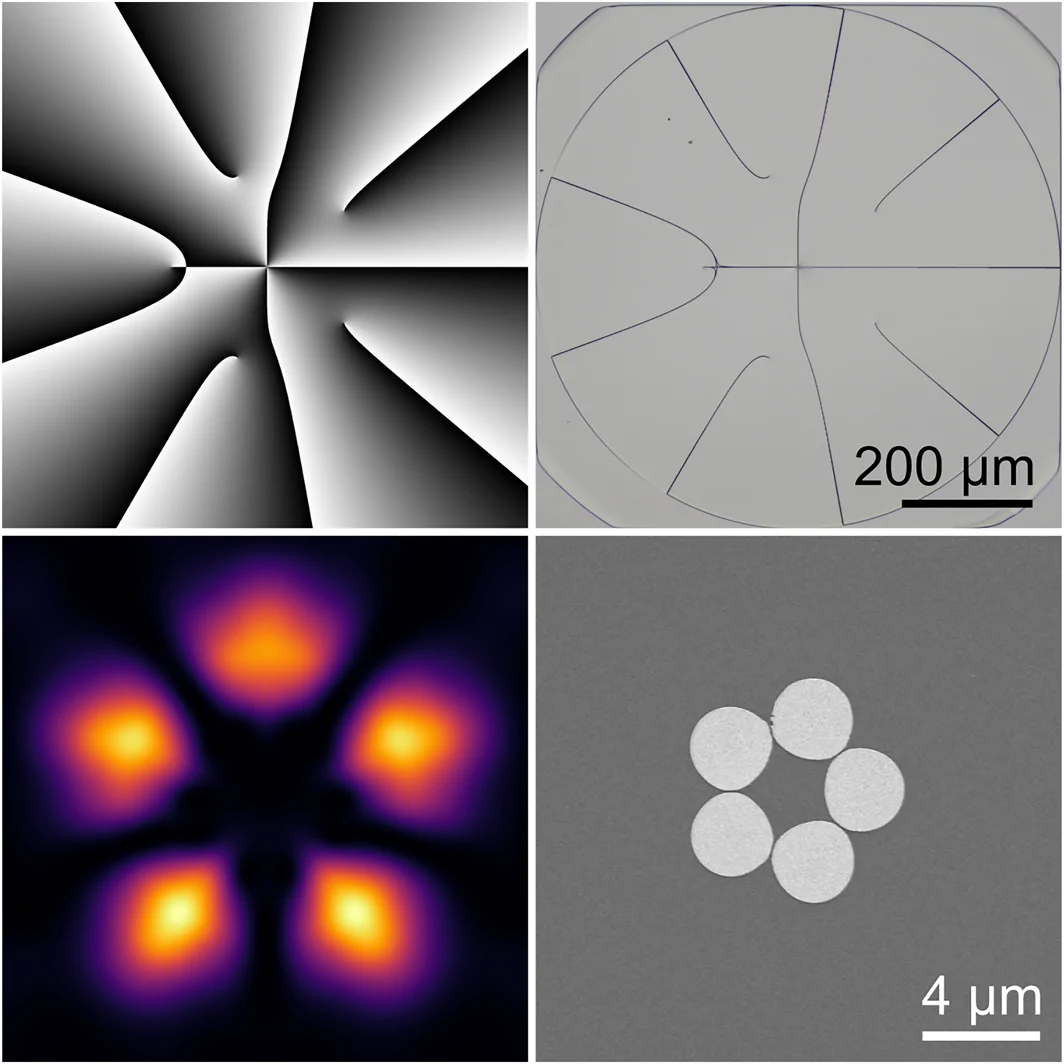

## 1 Introduction

Optical fields are characterized not only by their wavelength or polarization but also by the spatial distribution of phase, amplitude, and vectorial components across the beam profile. These transverse degrees of freedom constitute a vast and largely untapped parameter space when using conventional Gaussian beams [1]. Over the past 2 decades, the field of optics has increasingly focused on harnessing this spatial structure, giving rise to what is now broadly termed structured light [2]. A variety of approaches have been developed to impart tailored spatial phase or amplitude distributions onto optical beams, including liquid‐crystal spatial light modulators [3], digital micromirror devices [4, 5], and microfabricated geometric‐phase elements [6, 7]. These platforms have become standard tools for the laboratory generation of customized wavefronts. High‐precision 3D‐printed microoptics have recently emerged as a versatile and cost‐effective alternative, enabling complex diffractive elements with user‐defined geometries and extending the design space beyond conventional devices [8, 9, 10]. Complex light fields are of particular interest in the interaction of light and matter as many collective excitations or transitions are dipole forbidden and can thus not be directly excited. Complex light fields might be able to overcome this limitation and allow the study of formerly inaccessible transitions, for example, in complex plasmonic structures [11] or excitonic systems [12]. 3D printed masks are perfectly suited for a low‐cost and passive generation of such OAM light generation. Although an SLM would be more flexible in the designed phase mask, they can be quite expensive. Among the various forms of structured light, beams carrying orbital angular momentum (OAM) have attracted particular attention due to the unique rotational and topological properties they impart to the optical field [13, 14, 15, 16]. OAM beams facilitate a wide range of advanced functionalities, including multiplexed information transfer [17], optical trapping and manipulation of microscopic particles [18, 19], as well as enhanced imaging techniques such as edge enhancement or dark‐field‐like contrast [20]. Given their potential for high‐capacity information multiplexing in optical communication systems, efficient OAM detection has become a critical task. Previous approaches have demonstrated OAM detection using optical resonators [21] or coordinate transformation metasurfaces [22, 23, 24, 25]. In the context of nanophotonics, plasmonic nanogratings [26] and spin‐Hall couplers [27, 28] have been proposed for on‐chip OAM beam detection. The combination of structured light with nanophotonics, which studies light–matter interactions at the nanoscale, unlocks entirely new possibilities [29]. Nanophotonic systems have enabled breakthroughs that surpass the limitations of traditional photonics and electronics [30], including ultrafast, high‐bandwidth communication [31], and high‐capacity data storage [32, 33]. In addition, nanophotonics has provided transformative tools in biomedicine, ranging from novel diagnostic modalities [34] to light‐guided and light‐activated therapeutic techniques [35]. By integrating structured light with nanostructures, one gains unprecedented control over light at sub‐wavelength scales, enriching both fundamental studies and applied technologies.

This synergy motivates the present work, which focuses on generating complex structured light fields exclusively via 3D‐printed microoptics, specifically diffractive optical elements (DOEs), and investigating their interaction with sub‐micrometer gold oligomer structures by analyzing their absorptive behaviors. The study encompasses the full workflow: from the computer‐aided design of the diffractive elements, through generation and micrometer‐to nanometer‐scale focusing of the structured beams, to experimental and theoretical evaluation of their interactions with the nanostructures.

## 2 Theory

### 2.1 Beam Generation

The objective is to generate beams exhibiting intensity patterns composed of multiple bright lobes, with the number of lobes depending on the respective OAM quantum numbers. Such beams can, for instance, be realized through the superposition of two Laguerre–Gaussian (LG) modes with opposite azimuthal indices, that is, $l_{1}=-l_{2}$ [36]. LG modes are chosen as a natural basis as they form a complete set of solutions to the paraxial wave equation in cylindrical coordinates and thus inherently possess the rotational symmetry required to match the rotationally symmetric nanostructures investigated in this work. Pure LG modes, however, produce a rotationally symmetric ring‐shaped intensity profile whose radius grows with |l|, such that the extinction would depend only on the match between the ring radius and the radius of the circle on which the nanodisks are arranged, increasing with N but showing no symmetry‐dependent variation across beam modes. Superpositions of two LG modes are therefore employed to generate the azimuthally modulated, petal‐shaped intensity distributions required for symmetry‐selective interaction with the nanostructures. A single LG mode is defined as

(1) $$\begin{array}{ll} LG_{p}^{l}\left(r,\phi ,z\right) & =\frac{E_{0}}{w\left(z\right)}{\left(\frac{\sqrt{2}r}{w\left(z\right)}\right)}^{\left|l\right|}e^{-\frac{r^{2}}{w^{2}\left(z\right)}}\\ & \;\times L_{p}^{\left|l\right|}\left(\frac{2r^{2}}{w^{2}\left(z\right)}\right)e^{ikz+ik\frac{r^{2}}{R\left(z\right)}}\\ & \;\times e^{il\phi }e^{i\left(2p+|l|+1\right)\psi \left(z\right)}, \end{array}$$

where w(z) denotes the beam waist, p the radial index, $L_{p}^{\left|l\right|}$ the generalized Laguerre polynomial, R(z) the radius of wavefront curvature, and ψ(z) the Gouy phase [13]. This representation corresponds to the conventional definition of the LG mode, with the normalization factor set to unity. A direct combination of two OAM phase plates with transmission functions $e^{il_{1}\phi }$ and $e^{-il_{1}\phi }$ would, however, result in complete phase cancellation. To realize the intended multi‐lobed intensity structures, the concept must therefore be extended. As a starting point, we consider the superposition

(2) $$F\left(r,\phi \right)=LG_{p_{1}}^{l_{1}}+LG_{p_{2}}^{l_{2}}=E_{0}e^{-\frac{r^{2}}{w_{0}^{2}}}M\left(r,\phi \right),$$

evaluated in the beam‐waist plane (z=0). The *modulation factor* M(r,ϕ) is obtained by inserting the definition of the LG modes from Equation (1) into the superposition above, which leads to

(3) $$M\left(r,\phi \right)=R_{p_{1}}^{\left|l_{1}\right|}\left(r\right)e^{il_{1}\phi }+R_{p_{2}}^{\left|l_{2}\right|}\left(r\right)e^{il_{2}\phi },$$

where $R_{p_{i}}^{\left|l_{i}\right|}\left(r\right)$ denotes the real‐valued radial component of the LG mode without the Gaussian envelope. This procedure is formally analogous to a Gaussian input field that is modulated by a DOE. In such a scenario, however, simple phase modulation can only produce field distributions of the form:

(4) $$\hat{F}\left(r,\phi \right)=E_{0}e^{-\frac{r^{2}}{w_{0}^{2}}}e^{i\Phi \left(r,\phi \right)},$$

where Φ(r,ϕ) is the phase modulation. A comparison of $\hat{F}\left(r,\phi \right)$ with F(r,ϕ) shows that the required phase distribution corresponds to the complex argument of the modulation factor M(r,ϕ). This yields

(5) $$\Phi \left(r,\phi \right)=atan2\left(\frac{IM\left(r,\phi \right)}{RM\left(r,\phi \right)}\right),$$

where atan2(⋅) denotes the generalized inverse tangent function that ensures the correct evaluation of the phase within the interval (−π,π]. The corresponding far‐field intensity distribution associated with $\hat{F}\left(r,\phi \right)$ is obtained via the Fourier transform:

(6) $$\tilde{F}\left({\vec{k}}_{⊥}\right)=\underset{R^{2}}{\iint }\hat{F}\left(r,\phi \right)\;\tau \left(r,\phi \right)\;e^{-i{\vec{k}}_{⊥}\cdot \vec{r}}\;d^{2}\vec{r},$$

followed by computing its squared magnitude, that is, $I\left(r\right)\propto |\tilde{F}\left(\frac{2\pi r}{\lambda f}\right){|}^{2}$. The aperture transfer function τ(r,ϕ)=θ(f⋅NA−r), where θ(⋅) is the Heaviside step function, accounts for the finite numerical aperture NA=0.45 of the focusing microscope objective with a focal length of f=10mm. The beam has a waist of $w_{0}=0.44\;\text{mm}$ at the entrance of the microscope. Since the explicit numerical evaluation of this integral is computationally demanding, the Fourier transform is efficiently computed using a fast Fourier transform algorithm. To allow us to obtain unevenly many petals for the desired flower‐like intensity cross‐section, we move away from the condition $l_{2}=-l_{1}$ and instead identify the index combinations p=1 and $\left(l_{1},l_{2}\right)=\left\{\left(4,7\right),\left(4,8\right),\left(4,9\right)\right\}$ as producing the desired flower‐like beam structures, featuring $l^{\prime }=|l_{2}-l_{1}|=3,4,5$ bright petals in their intensity distributions, respectively. Although the number of petals is determined solely by $l^{\prime }$, the absolute values of $l_{1}$ and $l_{2}$ govern the radial intensity profile of the individual LG modes, see (1), and thus the radial position of the intensity maxima in the superposition beam. The choice of $l_{1}=4$ was made to produce petal radii matching the ring radius $r_{0}$ of the nanostructures. The corresponding wavefront phase patterns derived from these parameters, which simultaneously define the grayscale phase masks used for 3D printing, are shown in Figure 1a, together with the numerically calculated intensity profiles displayed in Figure 1c.

**FIGURE 1:**
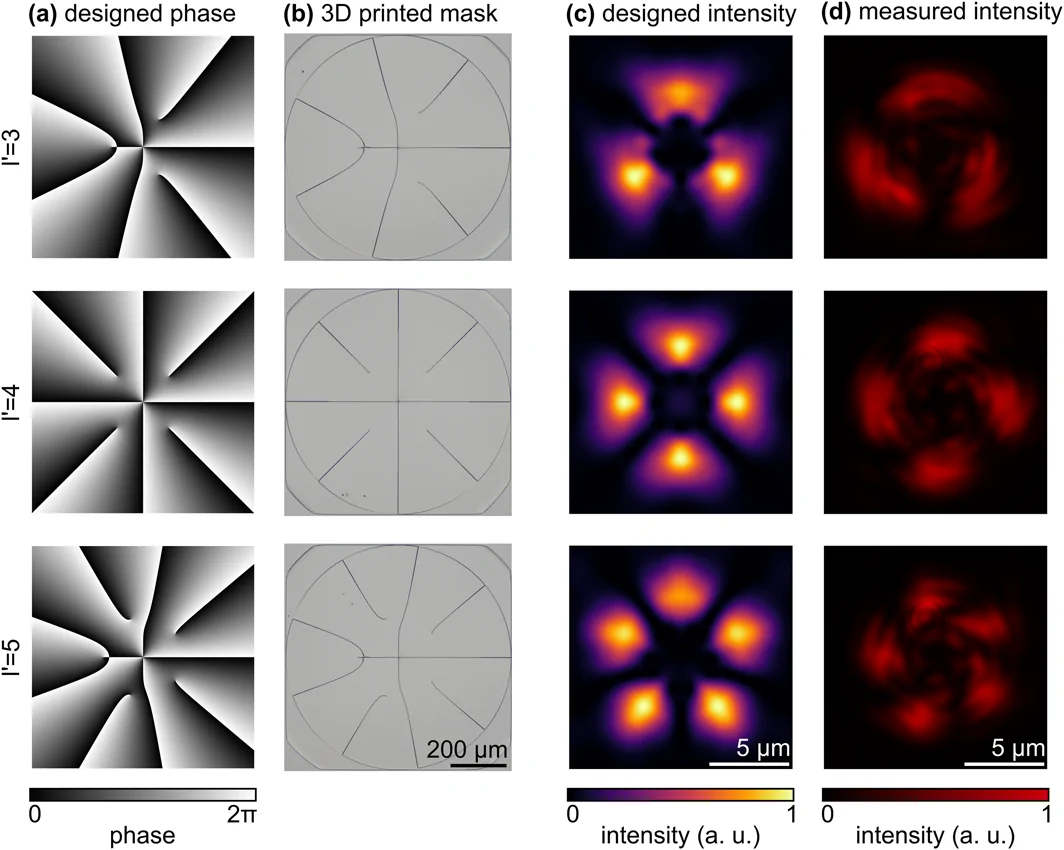
Phase masks and corresponding intensity distributions. (a) Designed grayscale phase. (b) Two‐photon polymerization grayscale lithography 3D printed transmission phase masks. The printed mask includes only a round cut‐out of the designed phase mask with a diameter of 800 μ m. (c) Designed intensity distribution in the focus of the objective. (d) Corresponding measured intensity distribution in the focus of the objective at a wavelength of 700 nm. The rows indicate the three different masks with $l^{\prime }\in \left\{3,4,5\right\}$. The scalebars in the lowest images of columns (b–d) apply to all images within the respective columns.

### 2.2 Interaction With Nanostructures

As the intensity distributions differ for these beam modes, it is reasonable to assume that the fraction of light absorbed by a given nanostructure depends on the mode order $l^{\prime }$. To quantify this behavior, we introduce the extinction coefficient:

(7) $$\eta_{\text{ext}}=1-\frac{P_{\text{tr}}}{P_{\text{tot}}}$$

which represents the fraction of light that is either absorbed or reflected, with $P_{\text{tot}}$ denoting the total incident power and $P_{\text{tr}}$ the transmitted power after interaction with the nanostructure. The investigated nanostructures consist of multiple gold nanodisks of radius R, symmetrically arranged on a circular ring of radius $r_{0}$. The number of disks is set to N=3,4,5, corresponding to the number of petals in the respective structured light beams. It is therefore anticipated that $\eta_{\text{ext}}$ reaches its maximum when the beam symmetry matches that of the nanostructure, that is, when $l^{\prime }=N$, as this condition yields optimal spatial overlap between the intensity maxima and the nanodisks. For other mode orders, the overlap decreases due to symmetry mismatch, resulting in lower extinction values. The fundamental Gaussian mode is expected to exhibit the highest transmission, since most of its intensity propagates through the central region between the nanodisks. Since $dP=I\left(r\right)\;d^{2}\vec{r}$, the total power of the incident light field is obtained by integrating the intensity distribution over the entire plane:

(8) $$P_{\text{tot}}=\underset{R^{2}}{\iint }I\left(r\right)\;d^{2}\vec{r}.$$

As all our structures do not show any resonant behavior, the intensity distribution of the incident light fields can be deduced and studied correctly even with the complex ring structures. Given the thickness of d=50nm of the gold nanodisks, the transmitted intensity can be neglected. Specifically, the skin depth of gold at the measurement wavelength of λ=700nm is approximately 13nm [37], such that the transmitted intensity is suppressed by a factor of $e^{-d/\delta }\approx 0.022$, which together with the high reflectance of approximately 0.971 of gold results in an expected transmittance on the order of $10^{-3}$. Therefore, the transmitted intensity through the metallic regions can safely be approximated as zero. Consequently, the total transmitted power is determined by integrating the intensity over the region excluding the absorbing areas:

(9) $$P_{\text{tr}}=\iint_{R^{2}\backslash C}I\left(r\right)\;d^{2}\vec{r},$$

where C denotes the total absorbing domain, defined as the union of N individual circular disks:

$$\begin{array}{ll} & C=\overset{N}{\underset{k=1}{⋃}}B_{k},\\ & \text{with}\;B_{k}=\left\{\left(x,y\right)\in R^{2}\;\left|\|\left(x,y\right)-M_{k}\|\leq R\right\}\right., \end{array}$$

and $M_{k}=\left(r_{0}\;\cos\frac{2\pi k}{N},r_{0}\;\sin\frac{2\pi k}{N}\right)$ denoting the center position of the k‐th disk. Since the disks are fabricated such that they do not overlap, that is, $\forall i\neq j:B_{i}\cap B_{j}=\emptyset$, the expression in Equation (9) can be simplified accordingly. In this case, the total absorbed power can be written as the sum of the contributions from the individual disks:

(10) $$P_{\text{abs}}=\sum_{k=1}^{N}\underset{B_{k}}{\iint }I\left(r\right)\;d^{2}\vec{r},$$

and the total transmitted power is obtained by subtracting this expression from the incident beam power defined in Equation (8).

## 3 Experimental Section

### 3.1 Setup

The experimental setup is shown in Figure 2. A supercontinuum white‐light laser (NKT Photonics SuperK Extreme), spectrally filtered to λ=700nm by an acousto‐optic tunable filter (NKT SuperK Select+, specified full width at half maximum 2.5–8.5 nm in the visible) is directed onto the 3D‐printed DOE. This phase mask is fabricated by two‐photon polymerization grayscale lithography on the Quantum X 3D printer (Nanoscribe GmbH & Co. KG). Representative microscope images of the fabricated structures are shown in Figure 1b. The fabrication process is described in more detail in the Appendix A and refs. [38, 39]. The round phase masks have a diameter of 800μm and are fabricated on a square pedestal with a side length of 800μm and a height of 5μm. This pedestal is cut off at large radii, due to the limited field of view of the writing objective and visible as rounded corners in Figure 1b, but does not impede optical performance. The generated beam is coupled into a 20× microscope objective with a numerical aperture of NA=0.45 and a focal length of f=10mm (Nikon CFI S Plan Fluor ELWD 20XC), which is mounted on a motorized z‐stage for precise axial positioning. It is tightly focused onto the nanostructures (see Appendix A for fabrication details), which are placed on a piezo‐stage in z‐direction (P‐736 PI nano Z) integrated into a x,y translation stage (Märzhäuser SCAN IM 130 × 85). The transmitted light is collected by the 60× imaging objective with NA=0.70 (Nikon CFI S Plan Fluor ELWD 60XC) and transmitted through the 1.5× tube lens for additional magnification. The resulting intensity distributions are recorded using a scientific camera (iDS U3‐31J0‐C‐HQ), whereas the transmitted optical power is measured with a power meter (Thorlabs S120C).

**FIGURE 2:**
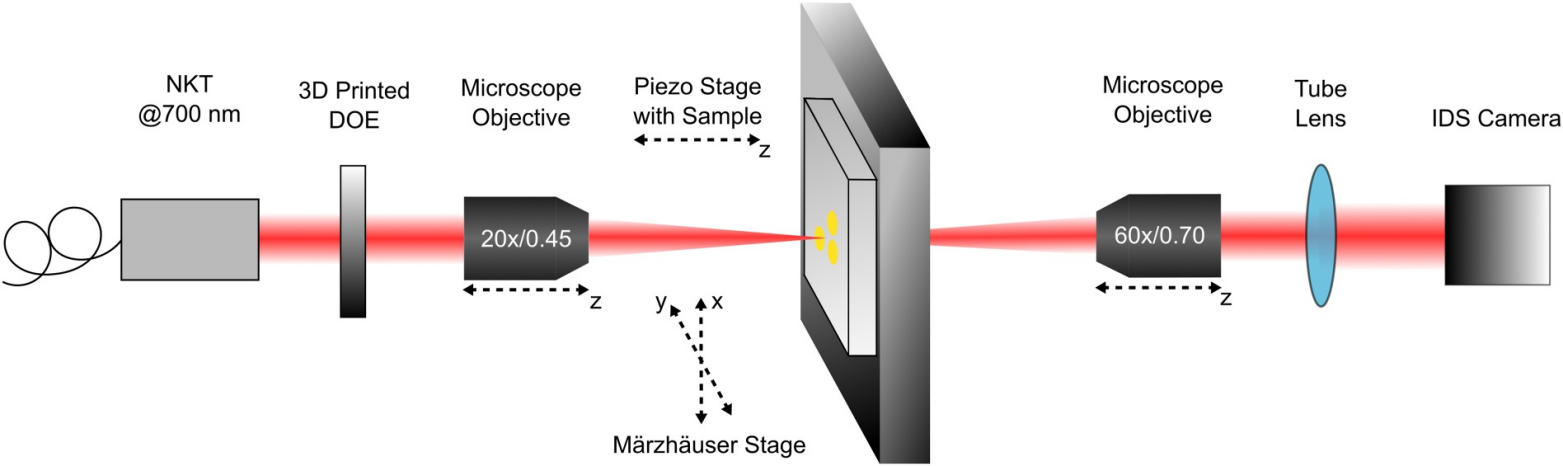
Schematic of the experimental setup. A supercontinuum white‐light laser by NKT Photonics is operated at a central wavelength of 700 nm (specified full width at half maximum 2.5–8.5 nm in the visible). The beam is shaped by a 3D‐printed DOE and subsequently focused by a microscope objective onto the sample, which is mounted on an *x*–*y* stage by Märzhäuser combined with a PI z‐piezo stage for precise positioning. The transmitted light is collected by an imaging microscope objective and relayed through a 1.5× tube lens onto a camera. For specific measurement procedures, the camera can be replaced by alternative detection devices, such as a power meter.

### 3.2 Measuring Method

To initially inspect the generated light fields, the setup is operated without the nanostructure sample and the camera is used for direct beam imaging. Afterward, the nanodisk arrays, which are arranged in a 20×6 matrix in which R varies from 200nm to 2μm in steps of 100nm across the columns and $r_{0}$ varies from 3.3μm to 4.3μm in steps of 200nm across the rows, are positioned precisely in the focal plane of the focusing microscope objective for all laser modes and for each disk configuration. Before the measurements were performed, the radial position of the intensity maxima of each structured light beam was characterized, and a single row is selected such that $r_{0}$ matches the beam radius. All subsequent measurements are performed within this row, ensuring that $r_{0}$ remains constant while R is varied across the columns. The total incident power is determined by laterally translating the sample out of the beam path and recording the resulting power with the power meter. The transmitted power is measured analogously with the nanostructure placed inside the beam. To achieve optimal spatial overlap between the petal‐shaped beam and the disk array, controlled rotation of the structured light field is required. This is accomplished by mounting the 3D printed DOEs in a θ‐rotation stage. When the number of petals in the beam matches the number of disks in the array, the alignment procedure becomes trivial: the beam is centered on the structure and their shared symmetry enforces a unique relative orientation, as shown in Figure 3a–c. Precise lateral alignment of the beam center with the center of the nanodisk array is critical, as any misalignment would shift the intensity maxima away from the nanodisks and into the gaps between them, thereby reducing the extinction significantly. If the number of petals and disks does not coincide, the rotational alignment must be defined explicitly to ensure comparability across all measurements. To remove this ambiguity, each petal‐shaped beam is aligned such that one of its intensity maxima coincides with a designated reference disk. This reference disk is always chosen to be the rightmost disk of the array, ensuring that the corresponding beam maximum lies along the positive x‐axis.

**FIGURE 3:**
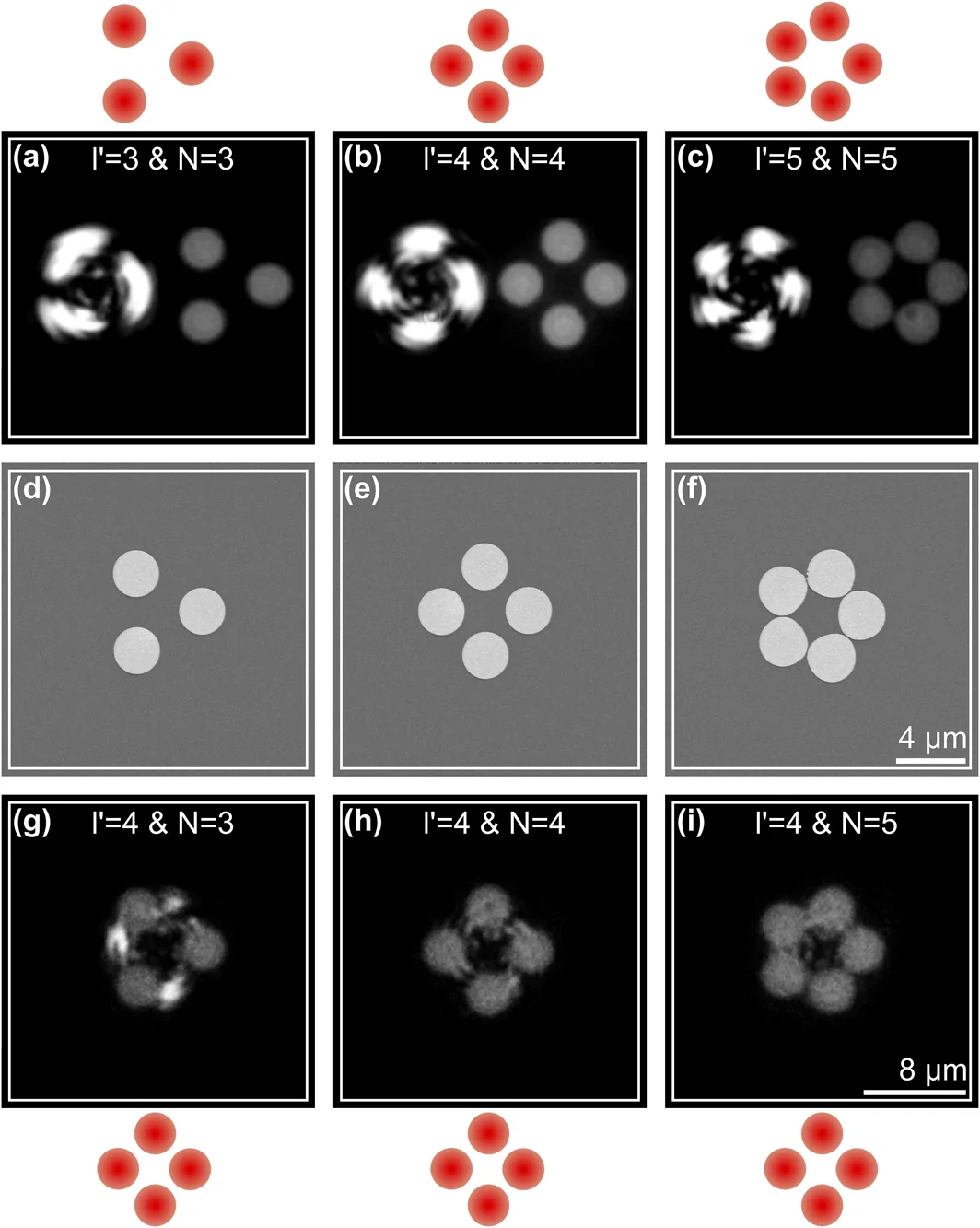
Images of the gold nanodisk arrays positioned in the focal plane of the laser beam are shown for the 3‐petal (a), 4‐petal (b), and 5‐petal (c) beam configurations. In each case, the nanodisk geometry is designed to match the corresponding beam symmetry and is placed adjacent to the recorded intensity pattern. SEM images of the gold nanodisks are shown in (d–f), where all disks have a radius of 2 μm and a thickness of 50  nm. Panels (g–i) display the spatial overlap between the 4‐petal beam and the 3‐, 4‐, and 5‐disk structures, respectively, illustrating the fraction of incident light obstructed by each configuration. Schematic representations between the images depict the corresponding nanostructures, whereas those positioned above and below the microscope images illustrate the beam profiles associated with the images beneath and above. The scale bar in panel (f) applies to all SEM images, whereas the scale bar in panel (i) applies to all remaining panels.

### 3.3 Evaluation Method

The beam intensity distributions recorded with the IDS camera require no additional processing aside from minor color adjustments and are shown in Figure 1d. To quantify the interaction with the nanostructures, the measured total and transmitted powers, $P_{\text{tot}}$ and $P_{\text{tr}}$, are converted into extinction coefficients using Equation (7) for both the experimental data and the theoretical model. The resulting extinction values are plotted for all investigated nanostructures, as displayed in Figure 4a–c for the three‐ to five‐petal configurations. To emphasize the differences more clearly, we highlight the nanostructure with a disk radius of R=1μm and present the corresponding extinction coefficients as adjacent colored bars as shown in Figure 4d–f. This visual arrangement allows for a direct and transparent comparison between experiment and theory. We would like to note that the disks as well as the overall ring arrangement do not show resonant behavior and can thus be modeled as purely ray‐optical elements. Thus, the geometric of the intensity maxima of the structured beam and the nanostructures is sufficient to describe the interaction. To further aid the analysis of the presented method, the mode purity could be investigated using a modal analysis of the structured light, such as explained by J. Pinnell et al. [40].

**FIGURE 4:**
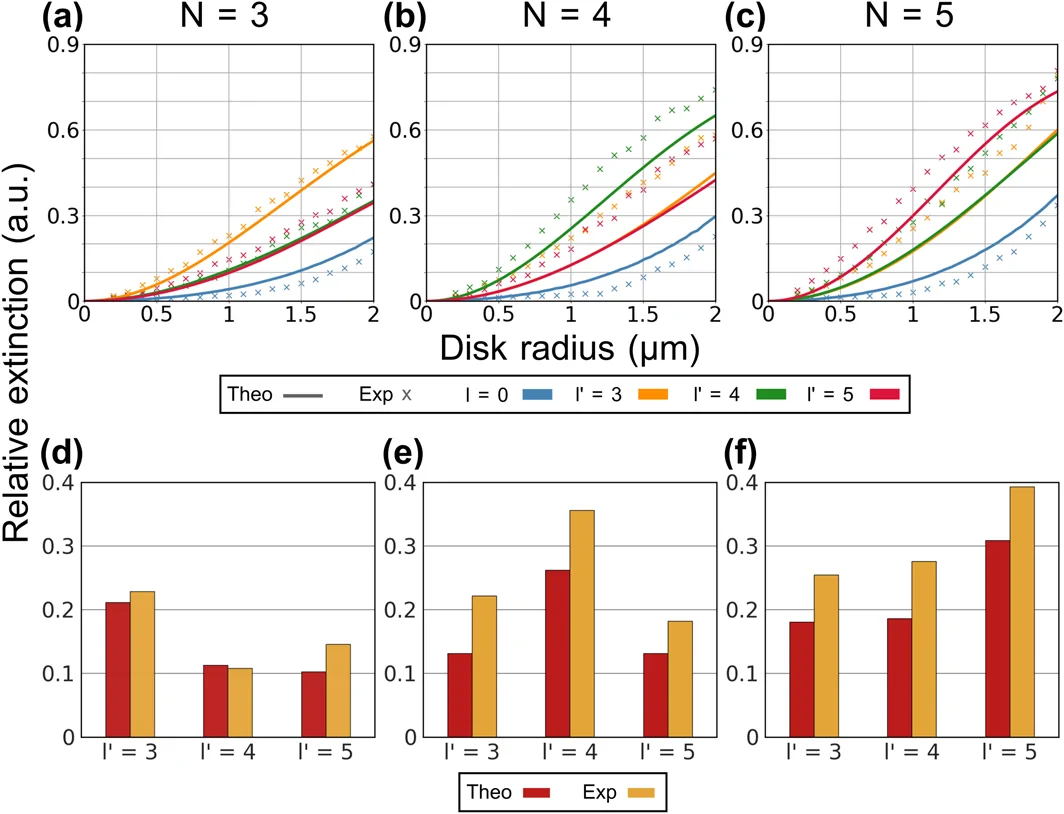
Experimentally measured relative extinction values for the $l^{\prime }=3,4,5$ petal‐shaped structured light beams are shown in orange, green, and violet, respectively, together with the Gaussian mode in blue. These results are compared with the corresponding theoretical predictions obtained from the spatial overlap integral as a function of the nanodisk radius R for nanostructures composed of N=3 (a), N=4 (b), and N=5 (c) gold disks, shown in the same colors. The bar plots below the curves display the extinction values for the N=3,4,5 structures in (d–f), respectively, for a disk radius of R=1μm. They highlight the overall extinction behavior and the deviations between the experiment (gold) and theory (red) for each nanostructure.

### 3.4 Data Discussion

The measured intensity distributions shown in Figure 1d are in good agreement with the numerically predicted profiles in Figure 1c, clearly reproducing the expected flower‐like structures with $l^{\prime }=3,4,5$ well‐distinguishable lobes, thereby confirming the validity of the beam generation approach. The individual lobes are of equal intensity by design, as the underlying phase pattern is derived from a symmetric superposition of LG modes with equal weights. The minor deviations from perfect lobe uniformity and shape observed in the experimental distributions are attributed to residual fabrication imperfections of the 3D‐printed DOEs, slight optical misalignments, and deviations of the input beam from an ideal Gaussian profile. A full phase characterization of the generated beams is not necessary for the present work, as the extinction measurement relies solely on the intensity distribution of the structured beam. The close agreement between the measured and numerically predicted intensity distributions therefore serves as sufficient confirmation that the DOEs imprint the intended phase patterns onto the input beam. For the nanostructure consisting of N=3 gold nanodisks, both the theory and experiment clearly show that the maximum extinction is obtained for the beam whose symmetry matches that of the structure, namely the $l^{\prime }=3$ mode shown in Figure 4a. This behavior is fully consistent with the expectation that two fields sharing a $C_{3}$‐symmetry exhibit an optimal spatial overlap when positioned in the configuration described above. The other structured light modes, corresponding to $C_{4}$‐ and $C_{5}$‐type intensity profiles, yield comparable extinction values and remain visibly below the $l^{\prime }=3$ mode, reflecting their weaker overlap with the three‐disk arrangement. As expected, the Gaussian mode produces the lowest extinction of all investigated beams, since its $C_{\infty }$‐symmetric intensity maximum lies at the center of the array and therefore transmits predominantly through the gap between the nanodisks rather than overlapping any of them. For decreasing disk radii, the extinction values decrease monotonically and approach zero for vanishingly small disk sizes, which is consistent with the fact that the absorbed power must vanish in the limit of zero absorbing area. Overall, the experimental data for N=3 reproduce the theoretical trends with high fidelity. The experimental data points generally lie slightly above the theoretical curves, indicating that the measured extinction is somewhat higher than the prediction obtained from Equation (7) combined with Equations (8) and (9). These small deviations suggest that additional physical effects not included in the current overlap‐based model may contribute, such as wave‐optical scattering or plasmonic interactions. The Gaussian beam constitutes an exception, as its experimental extinctions fall below the theoretical values, which can again be attributed to its fundamentally different field structure and its reduced sensitivity to scattering‐induced losses compared to the higher‐order modes. Most of these observations carry over directly to the N=4 and N=5 nanostructures as shown in Figure 4b,c. In both cases, the structured light mode whose intensity distribution shares the same rotational symmetry as the corresponding oligomer exhibits the highest extinction across all disk radii: the $l^{\prime }=4$ mode for the N=4 structure with its $C_{4}$‐symmetry and the $l^{\prime }=5$ mode for the N=5 structure with its $C_{5}$‐symmetry. The remaining $l^{\prime }$‐modes again display similar extinction values, reflecting their comparable overlap with the structures, whereas the Gaussian mode consistently provides the lowest extinction, in agreement with both the experiment and theory. This symmetry‐dependent blocking process is directly visualized in Figure 3g–i, where the high‐intensity lobes of the $l^{\prime }=4$ beam coincide optimally with the N=4 structure in panel (h) but exhibit reduced spatial overlap when incident on the N=3 (g) or N=5 (i) configurations. A notable trend becomes apparent when comparing the absolute extinction levels across different N: the extinction increases systematically with the number of disks. This behavior can be straightforwardly attributed to the enlargement of the absorbing area. As shown in Figure 3i, the N=5 structure occupies such a large portion of the circular array that it nearly approximates a continuous gold ring, thereby blocking a substantial fraction of the incident field for all $l^{\prime }$‐modes. At the same time, the deviations between the experiment and theory appear more pronounced for the N=4 and N=5 structures than for N=3, suggesting that the theoretical model captures fewer of the relevant physical effects as the symmetry order and structural complexity increase. The bar plots displayed in Figure 4g–i summarize the absolute extinction values for the structured light modes at a fixed disk radius of R=1μm for the N=3,4,5 structures, respectively.

The gold nanostructures are depicted in SEM images in Figure 3d–f. These plots clearly reinforce the trends discussed above. They demonstrate that the extinction is always highest when the symmetry of the beam matches that of the nanostructure, that the absolute extinction values increase systematically with the number of disks, and that the deviations between the experiment and theory remain comparatively small, consistently staying below 10 percentage points. This supports the validity of the theoretical assumptions while confirming the presence of only minor discrepancies consistent with the preceding discussion.

## 4 Conclusion and Outlook

This work establishes a solid foundation for advancing research at the interface of structured light and nanophotonics. We began by designing diffractive optical elements capable of generating complex structured light beams, fabricated via two‐photon polymerization grayscale lithography. Building on this, a mathematically rigorous framework was developed to construct complex phase distributions. Unlike approaches that reconstruct the phase from a target intensity profile, our method enables the direct generation of smooth, physically consistent phase patterns, facilitating the creation of multi‐lobed, flower‐like intensity distributions. When implemented in the optical path, these DOEs produced the targeted field structures upon focusing, in excellent agreement with semi‐analytical predictions. Experimentally, gold nanodisk oligomers with circular arrangements of varying numbers of disks were positioned in the focal plane of the structured beams and systematically scanned. The interaction was quantified via the extinction, obtained by comparing transmitted and incident powers. A theoretical model based on calculating the spatial overlap between the structured light fields and the gold nanodisks, while assuming negligible transmission through the disks, reproduced the experimental trends with high fidelity. Small deviations were observed, particularly for higher‐order structured light modes, suggesting that additional wave‐optical or plasmonic effects, not included in the present description, contribute to the measured response. These findings point to several promising directions for future research. On the theoretical side, incorporating plasmonic resonances, near‐field coupling, or more complete wave‐optical treatments would enable a more accurate description of the underlying light–matter interaction. Experimentally, reducing the illumination wavelength and achieving tighter focusing could allow direct probing of mode‐specific plasmonic responses. This would further benefit from employing smaller or more complex nanoparticle arrangements and expanding the illumination scheme beyond two‐dimensional field profiles. More broadly, the demonstrated principle of symmetry‐matched illumination is not restricted to circularly symmetric arrangements and extends in principle to arbitrarily shaped nanostructures, provided that a structured light field can be designed whose intensity distribution matches the spatial layout of the target geometry. In particular, the use of structured beams with engineered three‐dimensional field distributions offers a powerful route toward tailoring the interaction between light and nanoscale matter. Currently, the device is highly chromatic and offers only a limited working bandwidth. The bandwidth of the device could be enhanced using more intricate 3D printed optical elements [8] or using a different method to generate structured light [41]. The results of this work demonstrate both the feasibility and the versatility of combining structured light with nanostructured material systems, providing a robust platform for deeper fundamental investigations as well as for the development of practical applications in nanophotonics, photonics, and bio‐imaging. A particularly compelling direction for future research is the exploration of the spectral behavior of OAM or other structured beams interacting with plasmonic oligomers. Azimuthal and radial bonding and antibonding modes arising from plasmon hybridization [42] should give rise to distinct spectral absorption features, whose manifestation depends on the local polarization of the individual petals of the light structure [43].

## Author Contributions

**Noah Apostolico:** writing – original draft, writing – review and editing, formal analysis, software, methodology, validation, visualization, investigation, conceptualization, data curation. **Leander Siegle:** writing – original draft, writing – review and editing, conceptualization, investigation, visualization, validation, methodology, formal analysis, data curation. **Luca Schmid:** writing – original draft, writing – review and editing, conceptualization, investigation, visualization, validation, methodology, formal analysis, data curation. **Tim‐Dominik Gómez:** writing – original draft, writing – review and editing, software. **Julian Schwab:** writing – review and editing, writing – original draft, software. **Mario Hentschel:** conceptualization, funding acquisition, writing – review and editing, project administration, supervision, writing – original draft. **Harald Giessen:** conceptualization, funding acquisition, writing – review and editing, project administration, supervision, writing – original draft, resources.

## Funding

This work was supported by HORIZON EUROPE European Research Council (3DPRINTEDOPTICS: 862549); HORIZON EUROPE European Innovation Council (IV‐Lab: 101115545); Deutsche Forschungsgemeinschaft (DFG‐UP31/1,431314977/GRK 2642); Bundesministerium für Forschung, Technologie und Raumfahrt (Integrated3DPrint 13N16874, QLink.X, QR.X, QR.N); Ministerium für Wissenschaft, Forschung und Kunst Baden‐Württemberg (Innovation Campus Future Mobility: SdManu1, Lab7, Demo22); Baden‐Württemberg Stiftung (OPTERIAL); Carl‐Zeiss‐Stiftung (Center Qphoton, EndoPrint3D). J.S. acknowledges support from the Vector Stiftung (funding line “MINT‐Innovationen”), as well as from AvH Foundation.

## Conflicts of Interest

The authors declare no conflicts of interest.

## Figures and Tables

**FIGURE A1:**
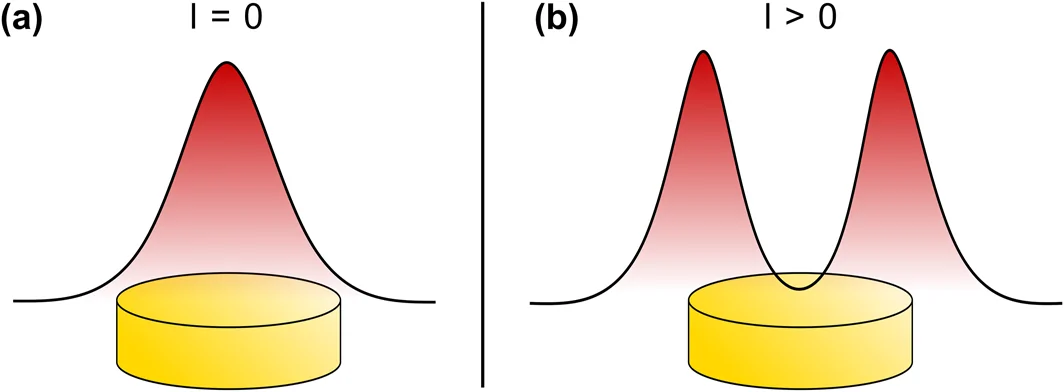
Illustration of a Gaussian beam (a) and a higher‐order OAM beam (b) interacting with a single gold nanodisk. The Gaussian beam exhibits its maximum intensity at the optical axis, resulting in strong extinction as the disk blocks the central high‐power region (dark red). In contrast, OAM beams feature a donut‐shaped intensity profile with power predominantly located in a ring around the axis, such that a smaller fraction overlaps with the disk, leading to reduced extinction.

## Data Availability

The data that support the findings of this study are available from the corresponding author upon reasonable request.

## Appendix A

### Fabrication

#### 3D Printed Transmission Phase Masks

The transmission phase masks depicted in Figure 1b are 3D printed by two‐photon polymerization grayscale lithography on the Quantum X 3D printer (Nanoscribe GmbH & Co. KG) with the photoresist Nanoscribe IP‐S (Nanoscribe GmbH & Co. KG) and a 25x/0.8 NA objective using the software GrayscribeX (Nanoscribe GmbH & Co. KG). Fabrication parameters of the phase masks include a slice distance of 1 μm, a hatch distance of 0.2 μm, and a scan speed of 200 mm/s. For writing, a laser power of 8–50 mW, a grayscale exponent of 1.459, and a multilayer attenuation of 0.8 are used. The fabrication process is described in more detail in refs. [38, 39]. The round phase masks have a diameter of 800 μm and are fabricated on a square pedestal with a side length of 800 μm and a height of 5 μm. This pedestal is cut off at large radii, due to the limited field of view of the writing objective and visible as rounded corners in Figure 1b, but does not impede optical performance. The relation between the topography z(x,y) of the phase masks in air (n≈1) and the phase Φ(x,y) illustrated in Figure 1a is given as

$$z\left(x,y\right)=\frac{\Phi \left(x,y\right)}{2\pi }\frac{\lambda }{n-1},$$

where λ is the wavelength of light that strikes the phase mask and n the refractive index of the phase mask material.

#### Gold Nanodisks

For the fabrication of the gold nanodisks, a cleaned borosilicate glass (Schott 263 T eco) substrate is spin‐coated with two layers of PMMA resist. The first layer is 200 K and the second layer is 950 K, both baked for 180 s at 150°C. For patterning in an electron‐beam lithography system (RAITH eLINE Plus), an additional layer of Espacer is used to avoid charging effects. After the resist development, a 3‐nm sticking layer of chrome is deposited on the substrate with a Pfeiffer Vacuum PLS 500 electron‐beam‐assisted evaporation system. On top of the sticking layer, 50 nm of gold is deposited, succeeded by the lift‐off in NEP.

### Analytical Far‐Field Intensity of a Fundamental OAM Beam

The scalar electric field of a Gaussian beam imprinted with the azimuthal phase factor $\Phi \left(\phi \right)=e^{il\phi }$ is given by

(A1)
$$E\left(r,\phi \right)=E_{0}e^{-\frac{r^{2}}{w_{0}^{2}}}e^{il\phi },$$

representing the most elementary form of an OAM beam. Using Equation (6) with τ(r,ϕ)=1 and evaluating the resulting Fourier integral, whose analytical treatment is notably cumbersome, the analytical expression for the far‐field intensity becomes

(A2)
$$\begin{array}{ll} I\left(r\right) & =I_{0}r^{2}e^{-\frac{{\left(2\pi \right)}^{2}w_{0}^{2}r^{2}}{4\lambda^{2}f^{2}}}\left[I_{\frac{1}{2}l-\frac{1}{2}}\left(\frac{{\left(2\pi \right)}^{2}w_{0}^{2}r^{2}}{8\lambda^{2}f^{2}}\right)\right.\\ & \;{\left.-I_{\frac{1}{2}l+\frac{1}{2}}\left(\frac{{\left(2\pi \right)}^{2}w_{0}^{2}r^{2}}{8\lambda^{2}f^{2}}\right)\right]}^{2} \end{array}$$

where $I_{\nu }$ denotes the modified Bessel function of the first kind of order ν. For l=0, the modified Bessel functions reduce accordingly and the upper equation simplifies to

(A3)
$$I\left(r\right)=I_{0}e^{-\frac{{\left(2\pi \right)}^{2}w_{0}^{2}r^{2}}{4\lambda^{2}f^{2}}},$$

recovering the well‐known Gaussian far‐field intensity distribution with its maximum located on the optical axis. For l>0, in contrast, the on‐axis intensity vanishes and the optical power is redistributed into an annular structure whose radial position increases with l. In the context of the extinction analysis presented in the main text, this redistribution of optical power governs the light–matter interaction with a nanodisk positioned on the optical axis. Figure A1 illustrates this simplest case: for a Gaussian beam, the disk blocks the central intensity, yielding high extinction, whereas for a higher‐order OAM mode, the annular intensity largely bypasses the disk, resulting in significantly reduced overlap and, consequently, a much lower extinction.

### Spot Size of a Fundamental OAM Beam

We seek to determine the diameter d of the donut‐shaped OAM beam, defined as the separation between the two radial intensity maxima. Since an analytical expression for the intensity distribution is available, see Equation (A2), the task reduces to locating the stationary points of I(r) with respect to r. Introducing the substitution $x=\frac{{\left(2\pi \right)}^{2}w_{0}^{2}r^{2}}{8\lambda^{2}f^{2}}$ and imposing $\frac{\partial I\left(x\right)}{\partial x}=0$ yields the transcendental condition

(A4)
$$0=I_{\frac{1}{2}l-\frac{1}{2}}\left(x\right)\left(l-4x\right)+I_{\frac{1}{2}l+\frac{1}{2}}\left(x\right)\left(l+4x\right)$$

after applying the recursive identity $\frac{2\nu }{x}I_{\nu }\left(x\right)=I_{\nu -1}\left(x\right)-I_{\nu +1}\left(x\right)$ together with the derivative relation $\frac{\partial I_{\nu }}{\partial x}=\frac{1}{2}\left(I_{\nu -1}+I_{\nu +1}\right)$. To obtain an approximate solution, we expand the modified Bessel functions in their Taylor series,

(A5)
$$I_{\nu }\left(x\right)=\sum_{m=0}^{\infty }\frac{1}{m!\;\Gamma \left(m+\nu +1\right)}{\left(\frac{x}{2}\right)}^{2m+\nu },$$

truncated after the first‐order term. This leads to the quartic equation:

(A6)
$$\begin{array}{ll} 0 & =\frac{1}{2}x^{4}-x^{3}\left[\frac{3}{8}l+\frac{3}{2}\right]+x^{2}\left[\frac{1}{8}l^{2}+\frac{11}{8}l+3\right]\\ & \;-x\left[\frac{3}{4}l^{2}+\frac{13}{4}l+3\right]+\frac{1}{4}l^{3}+l^{2}+\frac{3}{4}l \end{array}$$

whose analytical solutions follow from standard treatments of quartic equations, see ref. [44]. As including higher‐order terms in the Taylor expansion would increase the polynomial degree beyond four and render a closed‐form solution intractable, this represents the highest analytical accuracy attainable within this approach.
